# Von Hippel-Lindau Syndrome: An Updated Narrative Review for the First-Contact Clinician

**DOI:** 10.7759/cureus.112667

**Published:** 2026-07-14

**Authors:** Alan Alberto Pérez-Arzola, Israel Enrique Crisanto-López, Marelen Cruz-Cruz, Daniela Juárez-Melchor, Petra Yescas-Gómez, Juan Arriada-Mendicoa, Carlos Alberto Gómez-Pérez, Maximiliano Orozco-Pla, Miguel Ángel Ramírez-García

**Affiliations:** 1 Department of Genetics, National Institute of Neurology and Neurosurgery Manuel Velasco Suárez, Mexico City, MEX; 2 Reproductive Biology Laboratory, Mexican Social Security Institute, Puebla, MEX; 3 Department of Medical Genetics, General Hospital Zone No. 20, Mexican Social Security Institute, Puebla, MEX; 4 Department of Medical Genetics, Instituto Nacional de Pediatría, Mexico City, MEX; 5 Center for Health Sciences, University of Guadalajara, Guadalajara, MEX; 6 Department of Neurosurgery, National Institute of Neurology and Neurosurgery Manuel Velasco Suárez, Mexico City, MEX

**Keywords:** clear cell renal cell carcinoma, hemangioblastoma, review, vhl gene, von hippel-lindau syndrome

## Abstract

Von Hippel-Lindau (VHL) syndrome is an autosomal dominant (AD) condition that increases the risk of developing CNS and retinal hemangioblastomas (R-Hb), as well as clear cell renal cell carcinoma (ccRCC), pheochromocytomas, pancreatic neuroendocrine tumors (pNETs), and endolymphatic sac tumors. CNS hemangioblastomas and renal cell carcinomas are among the leading causes of mortality in affected individuals. Recent therapeutic advances, particularly hypoxia-inducible factor 2 alpha (HIF-2α) inhibitors and vascular endothelial growth factor (VEGF)-targeted therapies, have yielded promising results. However, active surveillance remains the cornerstone of clinical management for reducing associated morbidity and mortality. Because VHL syndrome is multisystemic, a multidisciplinary clinical team is necessary to provide comprehensive care, including targeted pharmacotherapy and surgical resection, to prevent severe complications. This narrative review outlines the current diagnostic approach and follow-up protocols for VHL syndrome, highlighting recent therapeutic advances.

## Introduction and background

Von Hippel-Lindau (VHL) syndrome is an inherited cancer predisposition syndrome with an autosomal dominant (AD) inheritance pattern. It is caused by pathogenic germline variants in the *VHL *tumor suppressor gene [1,2]. Briefly, the *VHL* gene plays an important role in regulating the cell cycle, angiogenesis, and apoptosis. Thus, variants that lead to loss of VHL protein function cause the accumulation of hypoxia-inducible factors (HIFs) and activate processes that promote cell growth and angiogenesis [3]. The term “VHL disease” was coined in 1936 to describe hereditary vascular tumors. von Hippel E first described the condition in 1904 as retinal angiomatosis [4], and Lindau A first described it in 1927 as a condition associated with cerebellar and spinal hemangioblastomas (Hb) [5,6].

VHL syndrome is considered a rare disease with high penetrance, approaching 100% by 60 years of age [2], and variable expressivity among affected individuals, including members of the same family who carry the same pathogenic variant. This phenotypic variability may be influenced by allelic heterogeneity, genetic modifiers, and environmental factors. VHL syndrome affects various organs and systems throughout life and predisposes affected individuals to the development of malignant and benign tumors [1,6,7]. Clinical manifestations of VHL include CNS-Hb, which occur in 70-80% of affected individuals [8], retinal hemangioblastomas (R-Hb), clear cell renal cell carcinoma (ccRCC), pheochromocytomas, pancreatic neuroendocrine tumors (pNETs), and endolymphatic sac tumors [3]. Patients with VHL syndrome may be diagnosed at different ages because each lesion has a distinct age of onset. Life expectancy has historically been reported to range from 40 to 60 years, with ccRCC and CNS-Hb being the main causes of mortality [9]. VHL syndrome is associated with substantial morbidity and mortality, underscoring the importance of molecular diagnosis and appropriate genetic counseling.

## Review

Epidemiology

The epidemiology of VHL syndrome varies among populations. Overall, the incidence is estimated to be one in 36,000 births [1]. European studies estimate a prevalence ranging from one in 38,951 to one in 91,111 people [8], and a 2019 study reported a prevalence of 2.13 per 100,000 people in the United States [10]. Approximately 20% of VHL cases result from de novo pathogenic variants, while the remaining 80% are familial [1]. The mean age of symptom onset is typically between the third and fourth decades of life [11].

The *VHL* gene and its role in etiopathogenesis

The *VHL* gene, located at locus 3p25-26, is a tumor suppressor gene. It consists of three exons and encodes a protein comprising 213 amino acids that is ubiquitously expressed [1,2]. The transcription patterns of the *VHL* gene are complex due to its cryptic exon (E1'), which results in different functional isoforms. There are two main isoforms: VHL30, which is named for its molecular mass (30 kDa), also known as VHL213 based on its amino acid length. VHL213 is the longest isoform. Another isoform is VHL19, which is named for its molecular mass (19 kDa), also known as VHL160 based on its amino acid length. These isoforms are biologically active and are expressed in all tissues [12]. The encoded protein participates in the ubiquitination and degradation of HIFs, which are transcription factors that regulate oxygen-dependent gene expression [13].

**Figure 1:**
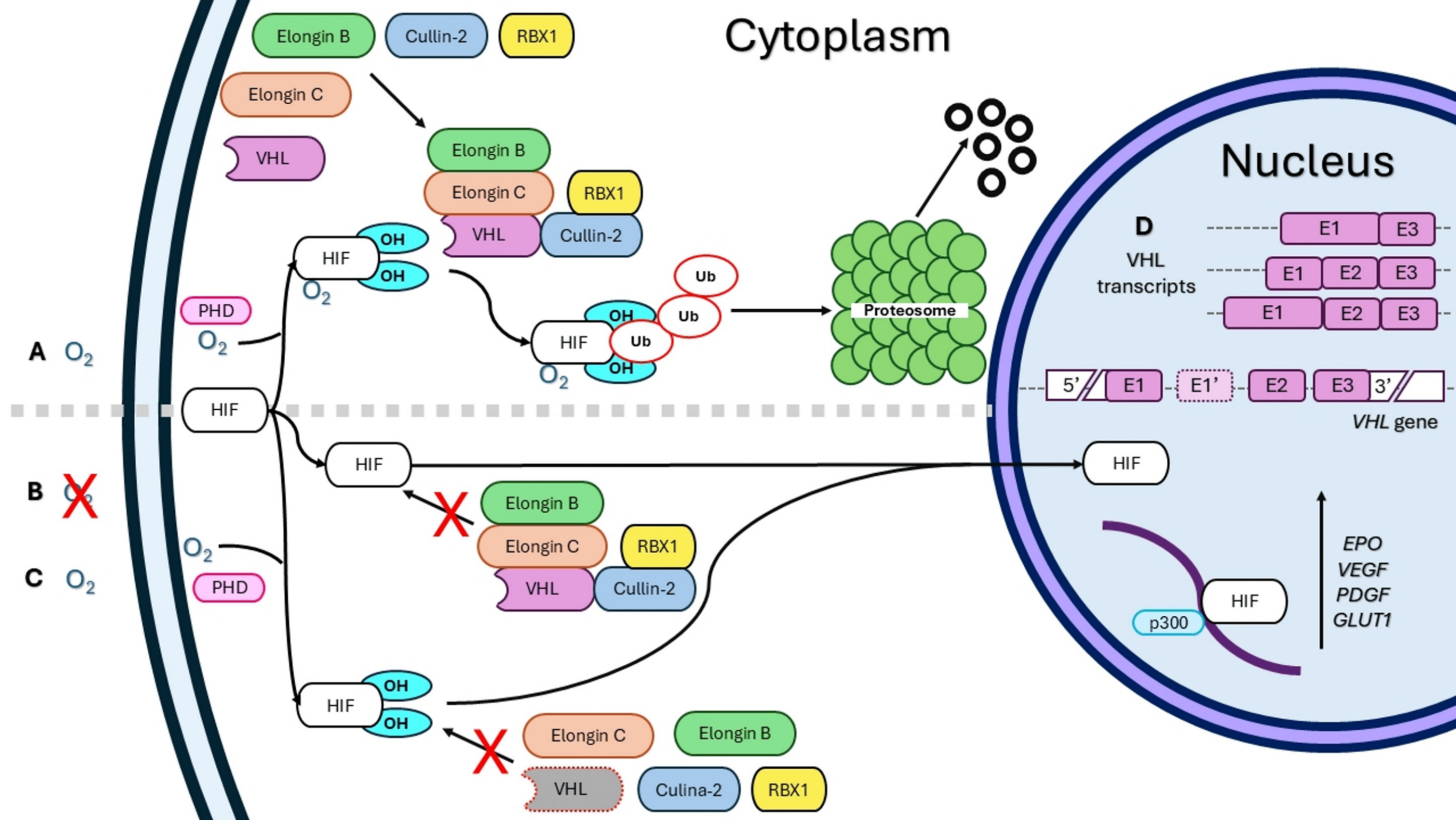
Cellular response to oxygen levels. In normoxia (A), oxygen is detected by hypoxia-inducible factors (HIFs), which are hydroxylated by the prolyl hydroxylase (PHD) superfamily. This allows the VHL complex to recognize the HIFs, which are then ubiquitinated and degraded by the proteasome. In hypoxia (B), however, the VHL complex cannot recognize the HIFs. Consequently, the HIFs translocate to the nucleus and activate genes such as *EPO*, *VEGF*, *PDGF*, and *GLUT1*, which are induced by hypoxia. If the VHL protein is altered, the VHL complex does not function properly. Consequently, it cannot ubiquitinate HIFs, allowing them to translocate to the nucleus and activate hypoxia-induced genes. Exon and splicing analysis of the *VHL* gene reveals a cryptic exon (E1′) that contributes to the formation of different transcripts (D). This image was created using Microsoft PowerPoint. VHL: von Hippel-Lindau; EPO: Erythropoietin; VEGF: Vascular endothelial growth factor; PDGF: Platelet-derived growth factor; GLUT1: Glucose transporter 1.

Proper folding of the VHL protein enables formation of the VBC complex with Elongin B, Elongin C, Cullin-2, and RBX1. The VBC complex is the substrate-binding subunit of the E3 ubiquitin ligase complex, which degrades HIFs, particularly HIF1α, HIF2α, and HIF3α [13]. The oxygen-dependent interaction between the VHL protein and HIFα is the primary mechanism by which mammalian cells detect and respond to hypoxia (Figure 1) [5,14].

Under normoxic conditions, HIF-α is hydroxylated at proline residues. This hydroxylated form is recognized by the VHL protein, leading to its rapid ubiquitination and degradation by the proteasome. However, under hypoxic conditions, prolyl hydroxylases become less active, resulting in the stabilization of HIF-α. Subsequently, HIF-α dimerizes with the constitutively expressed HIF-β protein and translocates to the nucleus. This dimeric form acts as a transcription factor that regulates the expression of various hypoxia-inducible genes involved in metabolism, glycolysis, angiogenesis, and apoptosis [15].

In addition, the VHL protein may participate in other cellular processes, such as DNA repair, JAK/STAT signaling [16], regulation of p53 activity [12], protein stabilization and phosphorylation, proteasomal degradation, microtubule stabilization, primary cilium formation, endocytosis, cytokine signaling, inflammation, apoptosis, and the regulation of senescence, among others [3,17].

VHL syndrome is caused by pathogenic germline variants in the *VHL* gene. According to Knudson's two-hit hypothesis, a second acquired somatic alteration is required. Specifically, one allele carries a germline pathogenic variant that results in loss of function, while the second allele undergoes an alteration in somatic cells that subsequently become tumor cells. This alteration may occur through a somatic point mutation, an extensive deletion, or promoter methylation [18].

Therefore, pathogenic variants in the *VHL* gene impair its function by reducing protein expression, causing protein misfolding, or promoting premature degradation. VHL protein deficiency can also activate oncogenic pathways [19]. Most variants are located in two functionally important regions: the HIF-1α-binding site, comprising amino acids 91-113, and the Elongin C-binding site, comprising amino acids 157-171 [15]. Abnormal VHL proteins prevent the degradation of HIF1α and HIF2α. This leads to their accumulation and increases the expression of VEGF, PDGF, EPO, CA9, and CXCR4, which play important roles in angiogenesis, cell proliferation, and metastasis [20,21].

Clinical manifestations

The clinical characteristics of VHL syndrome predominantly involve the development of benign and malignant neoplasms and cystic lesions affecting multiple organs. VHL syndrome includes CNS-Hb, R-Hb, ccRCC, pheochromocytoma, endolymphatic sac tumors, and cysts in the epididymis, broad ligament, pancreas, and kidneys [15,21-23]. In its early stages, VHL most commonly presents with CNS-Hb and R-Hb [24]. Three clinical types of VHL have been described based on genotype-phenotype correlations and the presence of ccRCC or pheochromocytoma [24,25].

Type 1 is the most common, accounting for 80% of cases. The associated variants result in a complete loss of function due to truncating or frameshift variants and large deletions. This leads to increased HIF expression, predisposing individuals to CNS-Hb, R-Hb, ccRCC, and other HIF-dependent neoplasms. However, pheochromocytoma rarely develops.

Type 2 is characterized by specific missense variants that tend to retain partial pVHL function and is subdivided as follows:

Type 2A includes CNS-Hb, R-Hb, and pheochromocytoma. This subtype has the best prognosis, with a low frequency of ccRCC and increased HIF expression.

Type 2B includes CNS-Hb, R-Hb, pheochromocytoma, pancreatic cysts, pNETs, and ccRCC. This type is distinguished by the presence of all the main manifestations, although this presentation is uncommon. Additionally, it exhibits increased HIF expression.

Type 2C selectively disrupts non-HIF functions and is marked by pheochromocytomas only.

A putative Type 3 has been described in the literature, although consensus is lacking. It refers to Chuvash polycythemia, which is caused by biallelic *VHL* gene variants and is characterized by elevated erythropoietin levels resulting from reduced HIF1 binding [24,26].

Unlike slow-growing tumors, which are usually asymptomatic, rapidly growing tumors become symptomatic and require resection. R-Hbs are usually the first manifestation of the disease, with an average age at diagnosis of 25 years. Approximately 90% of patients are affected by the age of 60 years [27]. These R-Hbs correspond to well-circumscribed, reddish, spherical vascular lesions in the peripheral retina or, occasionally, in the juxtapapillary area. They may be bilateral and multiple in 50%-60% of cases [28]. There are three growth patterns: exophytic, consisting of nodular, reddish, or orange lesions that grow in the outer layers of the retina; sessile, consisting of relatively flat, grayish, reddish, or orange lesions that develop in the middle layer of the retina; and endophytic, consisting of lesions that grow on the surface of the optic nerve or retina, between the inner limiting membrane and the inner plexiform membranes, and may protrude into the vitreous cavity [29]. If left untreated, they can cause visual impairment due to subretinal fluid accumulation or retinal traction [30].

CNS-Hbs affect 60%-80% of patients. These tumors grow slowly and multifocally, causing symptoms that vary depending on their location. The average age at diagnosis is 29 years, and these tumors are most frequently located in the cerebellum (45%-50%), spinal cord (35%-40%), and brainstem (2%-5%) [11,12]. Meanwhile, symptomatic medullary Hb develops at 47 years of age and is 1.5 to two times more prevalent in males [28,31].

Depending on its location, CNS-Hb can cause cranial hypertension due to tumor growth; cerebellar syndrome, including gait ataxia, dysmetria, headache, diplopia, vertigo, and vomiting; hypoesthesia, dysphagia, hyperreflexia, and headache when affecting the brainstem; or compressive syndromes in the spinal cord, with symptoms such as radiculopathy and myelopathy, including sensory deficits, proprioceptive changes, paraparesis, spinal hypertonia, hypoesthesia, weakness, gait ataxia, hyperreflexia, neurogenic pain, and incontinence [31]. However, CNS-Hb can remain asymptomatic for many years and behave benignly [12]. Rarely, CNS-Hb causes intraparenchymal or subarachnoid hemorrhage [29]. Although approximately 66% of CNS-Hbs occur sporadically, the remainder occur in the context of VHL syndrome and appear at an earlier age than in patients without the genetic condition [32,33]. Spinal cord tumors are rare, highly vascular, benign lesions that often present with syringomyelia. These tumors are associated with neurological morbidity [34]. Peritumoral cysts and syringes are the most common causes of symptoms, and 90% of patients with symptomatic CNS-Hb have associated syringes [35].

A tumor may develop in the vestibular aqueduct of the inner ear, which is located near the endolymphatic sac. This can result in dizziness, ear congestion, and hearing loss. These tumors are benign and occur in 4%-16% of patients with VHL syndrome. Hearing loss can occur through three mechanisms: direct erosion of the otic capsule, intralabyrinthine hemorrhage, and/or endolymphatic hydrops [36].

Approximately 70% of individuals with VHL syndrome develop solid or cystic pancreatic lesions. Although cystic lesions are usually benign, they can cause abdominal pain, vomiting, jaundice, weight loss, diarrhea, and, in rare cases, compression of the bile ducts. Between 9% and 17% of patients with VHL develop pNETs, which have a favorable prognosis because they are typically nonfunctioning and asymptomatic. However, larger tumors have metastatic potential [8,37,38].

Renal manifestations may present as cystic lesions in 50%-70% of cases. Between 40% and 80% of patients may develop ccRCC, which has a mean age at diagnosis of 39 years. ccRCCs may be bilateral and multiple, and they are the leading cause of mortality in VHL syndrome [10]. Sporadic ccRCC cases have been found to result from biallelic *VHL* inactivation. The *VHL* gene appears to act as a gatekeeper, and ccRCC mutations include loss-of-function mutations in *PBRM1*, *BAP1*, and *SETD2*; activation of the mTOR pathway through *PTEN* or *TSC1* mutations; and mutations that alter the response to DNA damage, such as *ATM* mutations [39].

Pheochromocytomas occur in 10%-20% of VHL syndrome cases. Patients are typically diagnosed at approximately 30 years of age. These tumors can be bilateral and multiple [12]. Pheochromocytoma is a rare neuroendocrine tumor of the adrenal medulla that causes excessive catecholamine secretion, leading to severe, potentially fatal hypertension and cardiovascular complications [40]. Diagnosis is based on biochemical tests, including plasma and urinary metanephrines; imaging studies, including CT and MRI; and functional imaging, including scintigraphy or PET [41].

The presence of other lesions depends on gender. In men, cysts and papillary cystadenomas may develop in the epididymis in 25%-65% of cases [42]. Bilateral cysts can lead to infertility due to obstructive azoospermia. These lesions are usually asymptomatic and are often discovered incidentally. When symptomatic, they present as slow-growing, painless scrotal swelling [43]. In women, broad ligament cystadenomas may occur, although less frequently. These benign lesions are managed conservatively because of their asymptomatic and nonprogressive nature [42]. These lesions can be diagnosed using CT imaging or ultrasound. They are also macroscopically and histologically similar to epididymal cystadenomas. If symptoms develop, surgery is necessary, and close monitoring is required because of their relationship with ccRCC and the associated risk of metastasis if the lesion increases in size [44]. Table 1 summarizes the main lesions of VHL syndrome.

**Table 1:** Principal lesions of von Hippel-Lindau syndrome, frequency among affected patients, and average age at diagnosis. VHL: von Hippel-Lindau; Hb: Hemangioblastoma; R-Hb: Retinal hemangioblastoma; CNS-Hb: Central nervous system hemangioblastoma; ccRCC: Clear cell renal cell carcinoma; pNETs: Pancreatic neuroendocrine tumors.

Lesion	Frequency or epidemiological characteristic	Average age at diagnosis, years
R-Hbs	90% of patients affected by age 60	25
CNS-Hb	60%-80% of patients	29
Symptomatic medullary Hb	1.5-2 times more prevalent in males	47
Endolymphatic sac tumors	4%-16% of patients	-
Solid or cystic pancreatic lesions	70% of patients	-
pNETs	9%-17% of patients	-
Renal cystic lesions	50%-70% of patients	-
ccRCC	40%-80% of patients	39
Pheochromocytomas	10%-20% of patients	30
Epididymal cysts/papillary cystadenomas	25%-65% of males	-
Broad ligament cystadenomas	Less frequent in females	-

Studies using neuroimaging techniques have identified Hb as a circumscribed lesion without a true capsule that exhibits either a solid or cystic growth pattern; tumors in the spinal cord are often associated with syrinx formation. CT scans reveal an isodense lesion relative to brain tissue that is typically devoid of calcifications. Hb lesions tend to appear as hypo- to isointense nodules on T1-weighted MRI sequences and as hyperintense nodules on T2-weighted sequences. These nodules contain serpentine flow voids within their nodular portions and in the area adjacent to the pia mater, creating a characteristic multiloculated or “popcorn” appearance [31]. Catheter angiography often reveals enlarged feeding arteries, dilated draining veins, and a dense central tumor blush. The best imaging technique for diagnosing Hb is contrast-enhanced MRI. Screening for VHL should include contrast-enhanced MRI of the entire neuroaxis [45]. From the age of 15 years, CNS MRI should be performed every two years. As soon as a CNS lesion is diagnosed, the frequency of CNS surveillance should be determined individually by the neurologist or neurosurgeon. In asymptomatic patients, two MRIs before the age of 15 years are recommended [37]. For pregnancy surveillance, contrast-enhanced MRI should be obtained before a planned pregnancy to characterize hemangioblastomas and/or hemangioblastoma-associated cysts before conception [9].

In terms of histological characteristics, Hb appears as solid and/or cystic masses at the macroscopic level. These masses are well circumscribed and surrounded by a pseudocapsule. Due to their high vascularity and lipid content, the cut surfaces appear red, yellow, or orange [29]. Microscopically, they exhibit compact, non-infiltrative growth patterns and variable lobularity. Because of their high vascularity, they contain large, branched vessels or areas of hemorrhage. Neoplastic stromal cells typically have clear, foamy cytoplasm containing lipid vacuoles. The nuclei exhibit slight pleomorphism, particularly in cases with degenerative atypia. These tumors have two main components: large neoplastic stromal cells with clear or foamy cytoplasm containing lipid vacuoles and abundant reactive vascular cells that form thin-walled vessels of variable sizes. Based on the predominant component, two histological variants are distinguished: reticular, with vascular predominance, and cellular, with stromal predominance [46].

Diagnosis of VHL syndrome

The clinical diagnosis of VHL syndrome is based on three main criteria: (1) CNS-Hb or R-Hb, (2) visceral lesions, and (3) family history. When there is a family history of VHL syndrome, the presence of one Hb or one visceral lesion confirms the diagnosis. However, in the absence of a family history, the presence of two or more Hb lesions, or one Hb lesion together with a visceral lesion, is required [1,47]. Table 2 describes the Dutch and Danish criteria for VHL syndrome.

**Table 2:** Published Dutch and Danish diagnostic criteria for VHL syndrome. VHL: von Hippel-Lindau; Hb: Hemangioblastoma; R-Hb: Retinal hemangioblastoma; CNS-Hb: Central nervous system hemangioblastoma; ccRCC: Clear cell renal cell carcinoma; pNETs: Pancreatic neuroendocrine tumors.

Published diagnostic criteria	Clinical diagnosis with a positive family history	Clinical diagnosis with no known family history	VHL-related manifestations included in both criteria	Manifestations specific to the Dutch criteria
Dutch criteria [38]	One VHL-related tumor and a first- or second-degree relative diagnosed with VHL syndrome	Two or more VHL-related manifestations	R-Hb; CNS-Hb; ccRCC; pheochromocytoma; pNET; endolymphatic sac tumor	Paraganglioma; multiple kidney cysts; multiple pancreatic cysts
Danish criteria [48]	One or more VHL-related manifestations and a first-degree relative diagnosed with VHL syndrome	Two or more Hbs, or one Hb and one VHL-related manifestation	R-Hb; CNS-Hb; ccRCC; pheochromocytoma; pNET; endolymphatic sac tumor	None

Next-generation sequencing (NGS) analysis detects approximately 85% of cases, while multiplex ligation-dependent probe amplification (MLPA) analysis detects approximately 10% of cases involving deletions or duplications. A study of VHL families found the following distribution of variants: 52% missense variants, 13% frameshift variants, 11% deletion variants, 11% nonsense variants, 7% splice-site variants, and 6% insertion/deletion variants [48]. Similar figures were observed in another recent study [49]. Biallelic variants in the *VHL* gene can cause different phenotypes, such as familial erythrocytosis type 2 or Chuvash polycythemia [50].

Treatment

Medical follow-up requires a multidisciplinary approach, and treatment must be tailored to each patient’s symptoms. Depending on the location, number, and morphology of the tumors, CNS-Hb usually requires surgical resection [3]. For spinal cord tumors, complete surgical resection is the preferred treatment [34]. Since cyst formation is a direct result of Hb, tumor resection often resolves the cysts [35]. Surgery is indicated if there is neurological deterioration related to the lesions or if the tumor and/or syringomyelia show progression, even if the patient is asymptomatic [51]. Lesion volume, location, and peritumoral edema have been suggested as factors associated with poor clinical outcomes. However, postoperative neurological prognostic factors are not well recognized [52].

The treatment options for R-Hbs range from clinical surveillance to thermal laser photocoagulation, photodynamic therapy, cryotherapy, radiotherapy, and vitreoretinal surgery [29,30]. The preferred treatment for endolymphatic sac tumors is surgical resection, which effectively controls the tumor and improves audiovestibular symptoms. Early detection allows for early resection, preventing hearing loss and reducing audiovestibular morbidity [36,37].

The management strategy for pNETs depends on prognostic factors such as functional status and tumor size. For instance, surgical resection is recommended for pNETs measuring 3 cm or more in the body or tail of the pancreas and 2 cm or more in the head. Additionally, tumor growth kinetics are considered [38]. For functional pNETs, symptom control is crucial and involves the use of drugs such as octreotide or lanreotide to inhibit hormone secretion and alleviate symptoms [1]. The primary treatment is surgical resection, ranging from partial pancreatectomy to more extensive resections. For metastatic or unresectable pNETs, tyrosine kinase inhibitors and mTOR inhibitors are used to control tumor growth. Small, low-grade tumors may require a partial pancreatectomy, whereas advanced, symptomatic tumors may necessitate a more aggressive surgical approach [38,39].

Management of pheochromocytoma involves controlling catecholamine levels with alpha-adrenergic blockers before surgery to prevent hypertensive crises [41]. This is followed by surgical resection of the tumor. For bilateral pheochromocytomas, laparoscopic or partial adrenalectomy may be considered to preserve adrenal function [3,40].

Serous cystadenomas are usually managed conservatively. Surgery is typically reserved for patients with significant symptoms or for those in whom a diagnosis cannot be established using other methods [38]. In particular, epididymal cystadenomas require conservative management and regular follow-up with imaging studies to monitor changes in size or the development of symptoms. Surgical intervention, including epididymectomy, may be considered if the tumor causes significant discomfort, grows rapidly, or is suspected to be malignant [43].

Targeted therapies

The standard treatment for ccRCC typically involves surgery, such as partial or total nephrectomy. However, the FDA has approved pazopanib, a multikinase angiogenesis inhibitor, for the treatment of advanced ccRCC. A single-arm phase II trial involving 31 individuals with a molecular or clinical diagnosis of VHL syndrome who were treated with oral pazopanib for 24 weeks reported that 13 of the 31 individuals (42%) achieved a response based on RECIST criteria after treatment. More than half (52%) of the individuals with ccRCC responded to therapy, including two individuals who achieved a complete response [53].

HIF and vascular endothelial growth factor (VEGF) inhibitors are also available. Belzutifan is a selective HIF-2α inhibitor that inhibits tumor growth by suppressing the transcriptional activity of HIF-2α in tumors and is used as a systemic treatment for VHL syndrome-associated ccRCC [3]. At a median follow-up of nearly 50 months, a single-arm phase II study reported that 41 (67%) of 61 participants with ccRCC had an objective response; seven (11%) had a complete response, and 34 (56%) had a partial response [54]. Belzutifan remains the only FDA-approved systemic therapy for VHL syndrome, although several next-generation HIF-2α inhibitors and combination strategies are currently under investigation [55].

Casdatifan (AB521) is a novel, potent allosteric HIF-2α inhibitor currently in clinical development. Preclinical studies have demonstrated that AB521 selectively inhibits HIF-2α-mediated transcription without affecting HIF-1α. When administered orally to mice, it caused dose-dependent decreases in HIF-2α pharmacodynamic markers and significant regression of human ccRCC xenograft tumors. AB521 also showed favorable activity when combined with cabozantinib, a tyrosine kinase inhibitor, and zimberelimab, an anti-PD-1 antibody, in ccRCC xenograft studies, suggesting its potential for combination therapy approaches [56].

NKT2152 is another HIF-2α inhibitor mentioned in recent literature as being investigated for potential therapeutic use, although detailed clinical data are limited. NKT2152 was evaluated in a phase I/II trial (NCT05119335) that enrolled 71 patients with ccRCC in the dose-escalation portion and 25 patients in a dose-expansion cohort. In a preliminary report, treatment-emergent adverse events were as expected and included anemia, hypoxia, and fatigue [57].

Small interfering RNA (siRNA)-based therapeutics targeting HIF-2α represent an emerging strategy for overcoming resistance mechanisms to small-molecule HIF-2α inhibitors. These RNA interference approaches offer an alternative mechanism for suppressing the HIF pathway and may expand therapeutic options beyond allosteric inhibitors [57,58].

Clinical and preclinical studies are also evaluating belzutifan in combination with cyclin-dependent kinase 4/6 inhibitors, tyrosine kinase inhibitors, and immune checkpoint inhibitors, including PD-1/PD-L1 antibodies. These approaches aim to enhance efficacy and potentially overcome resistance mechanisms [58,59]. Emerging resistance mechanisms to HIF-2α inhibitors include acquired mutations in the gatekeeper pocket of HIF-2α and mutations in the aryl hydrocarbon receptor nuclear translocator [57,58,60]. The development of next-generation HIF-2α inhibitors, such as casdatifan, along with combination strategies and alternative approaches, such as siRNA therapeutics, represents the current frontier in expanding treatment options beyond belzutifan for VHL disease.

Prognosis and follow-up

The prognosis of VHL syndrome depends on the age of onset, timing of diagnosis, baseline tumor volume, sex of the patient, and positive family history [61]. Early detection and intervention can prevent complications. Since 1990, surveillance guidelines have increased life expectancy by more than 16 years. Clinical follow-up includes ophthalmological examinations and biochemical tests for pheochromocytoma, such as measurements of catecholamines, metanephrines, normetanephrines, and vanillylmandelic acid. Audiometry is also performed [62]. Genetic testing can identify individuals with *VHL* gene variants before they develop symptoms and can detect carriers among family members, facilitating early surveillance [3].

As a result of early detection and more effective treatments, patients’ life expectancy and quality of life have improved. There is evidence that active surveillance reduces morbidity and mortality [1,3,9]. On average, patients have 8.5 tumors at the time of initial evaluation and develop 0.4 new tumors per year, with a higher frequency among younger individuals. *VHL* gene variants can be identified in peripheral blood or tumor tissue in familial cases but only in tumor tissue in sporadic cases [47].

Gadolinium-enhanced MRI is the most sensitive and specific method for monitoring CNS-Hb along the craniospinal axis. Lesions can be identified in individuals as young as eight years of age, and MRI surveillance is recommended to begin at age 11 years in symptomatic patients. During pregnancy, surveillance without gadolinium should be maintained. For individuals over 65 years of age with no prior history of CNS-Hb, imaging surveillance may be discontinued [9].

pNETs require continuous monitoring because of their metastatic and/or aggressive potential. Their prognosis depends on tumor grade, stage, and response to treatment. Early detection and comprehensive management are essential. Pancreatic serous cystadenomas should also be monitored using CT, MRI, or endoscopic ultrasound imaging [3,38].

In ccRCC, long-term follow-up is essential for detecting recurrence or metastasis. Metastasis of ccRCC to the adnexa and epididymis has been reported; therefore, these areas must be monitored. Pheochromocytomas have the potential to become malignant; therefore, a favorable prognosis depends on timely diagnosis and treatment, as well as the absence of other tumors [40]. Finally, although epididymal cystadenomas usually have a good prognosis, they require monitoring [44].

Genetic counseling

Genetic counseling is a communication process that involves providing patients and/or their family members with clear information about the inheritance, recurrence risks, and management options associated with VHL syndrome. It also involves consulting with patients and their families to determine appropriate follow-up and monitoring intervals. As previously mentioned, all patients with VHL syndrome should undergo CNS MRI surveillance with contrast media. Additionally, abdominal imaging is recommended around age 15 [9,61].

VHL syndrome is an autosomal dominant condition, meaning that patients have a 50% chance of passing it on to each of their children. It has almost complete penetrance and variable expressivity [1]. Additionally, intrafamilial phenotypic heterogeneity requires comprehensive, multidisciplinary surveillance to detect clinical manifestations early. Therefore, physicians and patients should be aware of the possibility of asymptomatic manifestations and allow for targeted surveillance of other organs [62].

## Conclusions

VHL syndrome is an AD condition that increases an individual's risk of developing hemangioblastomas of the CNS and retina, as well as clear cell renal cell carcinomas, pheochromocytomas, pancreatic neuroendocrine tumors, and endolymphatic sac tumors. The syndrome exhibits nearly complete penetrance and variable expressivity. Prognosis depends on the age of onset and the timing of diagnosis. Therefore, early detection and intervention can help prevent complications. Comprehensive management and follow-up should be provided by a multidisciplinary team, with an emphasis on surveillance to detect and treat tumors associated with VHL syndrome.
